# Factors influencing plasma concentration of voriconazole and voriconazole- N-oxide in younger adult and elderly patients

**DOI:** 10.3389/fphar.2023.1126580

**Published:** 2023-02-13

**Authors:** Lin Cheng, Zaiming Liang, Fang Liu, Ling Lin, Jiao Zhang, Linli Xie, Mingjie Yu, Fengjun Sun

**Affiliations:** Department of Pharmacy, The First Affiliated Hospital of Army Medical University, Third Military Medical University, Chongqing, China

**Keywords:** voriconazole, voriconazole-N-oxide, total bile acid, platelet count, estimated glomerular filtration rate, IL-6

## Abstract

**Background:** Voriconazole (VCZ) metabolism is influenced by many factors. Identifying independent influencing factors helps optimize VCZ dosing regimens and maintain its trough concentration (C_0_) in the therapeutic window.

**Methods:** We conducted a prospective study investigating independent factors influencing VCZ C_0_ and the VCZ C_0_ to VCZ N-oxide concentration ratio (C_0_/C_N_) in younger adults and elderly patients. A stepwise multivariate linear regression model, including the IL-6 inflammatory marker, was used. The receiver operating characteristic (ROC) curve analysis was used to evaluate the predictive effect of the indicator.

**Results:** A total of 463 VCZ C_0_ were analyzed from 304 patients. In younger adult patients, the independent factors that influenced VCZ C_0_ were the levels of total bile acid (TBA) and glutamic-pyruvic transaminase (ALT) and the use of proton-pump inhibitors. The independent factors influencing VCZ C_0_/C_N_ were IL-6, age, direct bilirubin, and TBA. The TBA level was positively associated with VCZ C_0_ (*ρ* = 0.176, *p* = 0.019). VCZ C_0_ increased significantly when the TBA levels were higher than 10 μmol/L (*p* = 0.027). ROC curve analysis indicated that when the TBA level ≥4.05 μmol/L, the incidence of a VCZ C_0_ greater than 5 μg/ml (95% CI = 0.54–0.74) (*p* = 0.007) increased. In elderly patients, the influencing factors of VCZ C_0_ were DBIL, albumin, and estimated glomerular filtration rate (eGFR). The independent factors that affected VCZ C_0_/C_N_ were eGFR, ALT, γ-glutamyl transferase, TBA, and platelet count. TBA levels showed a positive association with VCZ C_0_ (*ρ* = 0.204, *p* = 0.006) and C_0_/C_N_ (*ρ* = 0.342, *p* < 0.001). VCZ C_0_/C_N_ increased significantly when TBA levels were greater than 10 μmol/L (*p* = 0.025). ROC curve analysis indicated that when the TBA level ≥14.55 μmol/L, the incidence of a VCZ C_0_ greater than 5 μg/ml (95% CI = 0.52–0.71) (*p* = 0.048) increased.

**Conclusion:** TBA level may serve as a novel marker for VCZ metabolism. eGFR and platelet count should also be considered when using VCZ, especially in elderly patients.

## Introduction

Invasive fungal infections (IFIs) remain a clinical problem with high morbidity and mortality despite recent advances in diagnosis and treatment (Jenks et al., 2020). Common pathogens of IFIs are *Candida*, *Cryptococcus*, *Aspergillus*, and *Mucormycetes*. Except for patients with underlying hematologic malignancies, solid organ transplant recipients, and critically ill patients, high rates of IFIs and mortality are also observed among patients 65 years or older (Vallabhaneni et al., 2017; Matthaiou et al., 2018; Hesstvedt et al., 2019; Tsay et al., 2020). Voriconazole (VCZ) is an essential drug for treating IFIs, especially those caused by *Aspergillus* and *Candida*. It is a first-line therapy for patients with invasive *Aspergillosis* (Ullmann et al., 2018). However, VCZ has a narrow therapeutic range. A trough level of 1–5.5 mg/L is recommended for most European patients on VCZ prophylaxis or treatment (Ullmann et al., 2018), while a range of 0.5–5 mg/L is considered adequate for Chinese patients (Chen et al., 2018). Maintaining VCZ trough concentration (C_0_) in the therapeutic range is crucial in enhancing its treatment effect.

VCZ exhibits non-linear pharmacokinetics with large interindividual and intraindividual variabilities (Purkins et al., 2002; Theuretzbacher et al., 2006). Many factors influence VCZ C_0_, such as age, sex, VCZ dose and administration route, albumin, total bilirubin (TBIL), glutamic-pyruvic transaminase (ALT), glutamic-oxalacetic transaminase (AST), γ-glutamyl transferase (γ-GT), CYP2C19 gene polymorphisms, and inflammatory state. (Vanstraelen et al., 2014; Niioka et al., 2017; Veringa et al., 2017). However, the specificity of each index has certain limitations. Both clinical symptoms and test results must be considered to diagnose and treat infectious diseases. VCZ dosing regimens also require modification according to patients’ conditions.

Our previous study found that VCZ C_0_ in elderly patients was significantly higher than in younger adult patients. The proportion of patients with C_0_ greater than 5 mg/L was higher in older adults (Cheng et al., 2020). VCZ C_0_ in elderly patients was not significantly affected by CYP2C19 polymorphisms (Shang et al., 2020). Inflammation could affect liver function, C_0_, and the concentration ratio of VCZ C_0_ to VCZ N-oxide (C_0_/C_N_) in younger and older patients (Liang et al., 2022). Therefore, disease state and patient status could confer significant dynamic markers that contribute to the fluctuation of VCZ concentrations (Chantharit et al., 2020).

According to the US Food and Drug Administration Adverse Event Reporting System (2004–2021 data), the VCZ-induced liver injury ratio is 32.45% (Zhou et al., 2022). Intrinsic and idiosyncratic drug-induced hepatotoxicity causes alterations in bile acid homeostasis (Mosedale and Watkins, 2017). Thus, the total bile acid (TBA) level can influence VCZ metabolism. Platelets are key effector cells for inflammatory responses and have particular advantages (Jenne et al., 2013). Platelet count was one of the determinants of VCZ C_0_ in kidney transplant recipients (Zhao Y. C. et al., 2021). VCZ clearance was also significantly associated with platelet count in patients with liver dysfunction (Tang et al., 2019; Tang et al., 2021). The worsening of renal function was significantly associated with a cumulative dose of intravenous VCZ (≥400 mg/kg) (Yasu et al., 2018). Elderly patients often have impaired liver function, renal function, and chronic inflammation induced by chronic disease conditions. Therefore, we hypothesized that platelet count and renal function might affect VCZ metabolism in elderly patients.

This study aimed to identify the factors affecting VCZ C_0_ and C_0_/C_N_ in younger adults and elderly patients using the stepwise multivariate linear regression model. In addition to the influencing factors reported in the literature, the TBA, IL-6, platelet count, hemoglobin, and renal function indexes were also included in the study.

## Materials and methods

### Patients and study design

A single-center prospective study was conducted from January 2018 to June 2022. The study analyzed patients who received both VCZ prophylaxis and treatment. The inclusion criteria were patients who: (a) received VCZ therapeutic drug monitoring (TDM); (b) aged ≥18 years; (c) with steady-state VCZ C_0_ ≥ 0.4 μg/ml; (d) with available IL-6 concentration data measured on the same day of VCZ C_0_ measurement (IL-6 level was routinely detected in our hospital); (e) with available routine blood, liver function, and renal function results measured on the same day of VCZ C_0_ measurement; and (f) agreed to the use of their blood samples for VCZ C_N_ determination and signed informed consent forms.

This study was approved by the Ethics Committee of the First Affiliated Hospital of the Army Medical University. Patients were divided into two cohorts according to age: the elderly cohort (≥60 years) and the younger adult cohort (<60 years).

### Data collection

The following data were collected from the medical chart: (a) demographic and clinical characteristics, including age, sex, weight, underlying diseases, fungal infection, VCZ dose and administration route, and combined use of proton-pump inhibitors (PPIs); (b) inflammation marker IL-6 levels; (c) routine blood examination indices, including hemoglobin levels and platelet count; (d) liver function indices, including alkaline phosphatase (ALP), ALT, AST, γ-GT, TBA, albumin, TBIL, direct bilirubin (DBIL), and indirect bilirubin (IBIL) levels; and (e) renal function indices, including urea nitrogen, creatinine levels, and estimated glomerular filtration rate (eGFR). VCZ dosing was adjusted according to the TDM result at the VCZ C_0_ measurement.

### VCZ C_0_ and VCZ C_N_ determination

VCZ C_0_ was measured routinely in the clinic. The steady state of VCZ C_0_ was defined as the concentration obtained after 3 days of intravenous VCZ therapy (a loading dose of 6 mg/kg) or oral VCZ therapy (a loading dose of 400 mg) or the concentration obtained after 5 days of VCZ therapy without a loading dose. VCZ N-oxide is the primary metabolite in plasma, accounting for 72% of circulating VCZ metabolites (Geist et al., 2013). The plasma VCZ C_0_/C_N_ ratio may provide information about VCZ clearance. Therefore, the VCZ C_N_ was detected. VCZ C_N_ was measured together with VCZ C_0_ using liquid chromatography-tandem mass spectrometry (LC-MS/MS) as previously described (Shang et al., 2020). The limit of detection (LOD) of VCZ and VCZ N-oxide was 8 ng/ml and 10 ng/ml, respectively. The lower limit of quantification (LLOQ) of VCZ and VCZ N-oxide were both 400 ng/ml.

### Statistical analysis

IBM SPSS 19.0 (IBM Corp., Armonk, NY, USA) was used to perform the analysis. Categorical data were compared with the chi-square test. Data that do not conform to a normal distribution are represented by the median and interquartile range (IQR). Data from the two cohorts were compared using independent sample t-tests and Mann-Whitney U tests. A stepwise multivariate linear regression model was used to identify the factors influencing the VCZ C_0_ and C_0_/C_N_ ratios.

A total of 20 factors were used in the analysis, including sex, age, route of administration of VCZ, VCZ dose, combined use of PPIs, platelet count, and levels of hemoglobin, ALP, ALT, AST, γ-GT, TBA, albumin, TBIL, DBIL, IBIL, urea nitrogen, creatinine, eGFR, and IL-6. Additionally, the Spearman correlation test was performed to assess the association of the TBA level with VCZ C_0_ and VCZ C_0_/C_N_. The receiver operating characteristic (ROC) curve analysis was used to evaluate the predictive effect of indicators. Covariates with a *p*-value < 0.1 in the univariate analysis were entered into the multivariate analysis. A *p*-value < 0.05 was considered statistically significant.

## Results

### Younger adult patients

A total of 161 younger adult patients were included, with 229 VCZ C_0_ and 102 VCZ C_0_/C_N_. The primary baseline diseases were pneumonia, kidney disease, and leukemia. Almost a third of the patients had negative fungus detection results. Most patients received VCZ intravenously, with a dose of 3.6 ± 0.9 mg/kg, twice daily. Almost half of the patients received PPIs when taking VCZ (Table 1). The percentages of ALP, ALT, AST, TBA, albumin, TBIL, and DBIL within the normal range were 58.3%, 53.4%, 50.0%, 75.7%, 69.3%, 71.9%, and 61.6%, respectively (Table 2).

**TABLE 1:** Demographic and clinical chacteristics of patients in the two cohorts.

Variable	Younger adult cohort (n = 161)	Elderly cohort (n = 143)	*p*-value
Sex	—	—	0.001
Male (n [%])	96 (59.6)	110 (76.9)	—
Female (n [%])	65 (40.4)	33 (23.1)	—
Age (y)	43 ± 12	72 ± 8	<0.001
Underlying diseases	—	—	—
Leukemia (no. [%])	44 (27.3)	9 (6.3)	—
Hypertension (no. [%])	40 (24.8)	59 (41.3)	—
Diabetes mellitus (no. [%])	23 (14.3)	44 (30.8)	—
Coronary heart disease (no. [%])	2 (1.2)	18 (12.6)	—
Kidney disease (no. [%])	69 (42.9)	53 (37.1)	—
Pneumonia (no. [%])	97 (60.2)	113 (79.0)	—
Fungus category			—
Aspergillus (no. [%])	29 (18.0)	36 (25.2)	—
Saccharomycetes (no. [%])	14 (8.7)	21 (14.7)	—
Monilia (no. [%])	26 (16.1)	33 (23.1)	—
Unidentified fungi (no. [%])	30 (18.6)	27 (18.9)	—
Others (no. [%])	5 (3.1)	1 (0.7)	—
Negative (no. [%])	58 (36.0)	34 (23.8)	—
Route of administration	—	—	0.115
Intravenous (n [%])	130 (80.7)	125 (87.4)	—
Oral (n [%])	31 (19.3)	18 (12.6)	—
VCZ dose (mg/kg/dose)	3.6 ± 0.9	3.4 ± 0.9	0.023
Use of PPI	84 (52.2)	80 (55.9)	0.510

A patient may have several underlying diseases or fungus categories. Abbreviations: PPI, proton-pump inhibitor.

**TABLE 2:** Laboratory data of patients in the two cohorts.

Variable	Younger adult cohort (*n* = 229)	Elderly cohort (*n* = 234)	*p*-value
Voriconazole C_0_ (0.5–5.0 μg/ml)	3.00 (1.60, 4.81)	3.64 (2.12, 5.50)	0.027
Voriconazole C_0_/C_N_	1.33 (0.67, 3.11)	1.85 (0.85, 3.23)	0.307
IL-6 (0–7 ng/L)	23.9 (6.1, 75.0)	39.9 (15.9, 106.8)	0.001
Platelet count (125–350 ×10^9^/L)	113 (39, 211)	169 (96, 255)	<0.001
Hemoglobin (115–150 g/L)	87.6 ± 19.3	93.8 ± 19.0	0.001
Liver function			
ALP (38–126 U/L)	100.0 (73.5, 147.9)	114.7 (80.0, 159.0)	0.170
ALT (13–69 U/L)	22.0 (10.4, 46.0)	22.0 (12.0, 37.9)	0.542
AST (15–46 U/L)	30.9 (18.3, 57.0)	36.8 (24.1, 54.1)	0.068
γ-GT (12–58 U/L)	69.4 (35.0, 151.2)	86.5 (43.8, 169.6)	0.082
TBA (0–10 μmol/L)	4.4 (2.2, 9.8)	5.6 (3.0, 12.0)	0.015
Albumin (30–50 g/L)	33.0 ± 5.6	33.1 ± 5.1	0.870
TBIL (3–22 μmol/L)	13.0 (8.9, 23.8)	14.1 (9.8, 22.7)	0.333
DBIL (0–6 μmol/L)	3.8 (1.7, 8.7)	4.6 (2.5, 10.2)	0.069
IBIL (3–16 μmol/L)	8.7 (5.9, 14.0)	9.1 (6.8, 12.9)	0.436
Renal Function			
Urea nitrogen (1.7–8.3 mmol/L)	9.2 (6.0, 14.1)	12.6 (5.5, 20.0)	0.032
Creatinine (59–104 μmol/L)	81.2 (52.2, 151.5)	75.2 (53.8, 126.0)	0.555
eGFR (80–120 ml/min)	91.7 (41.0, 119.7)	83.9 (46.1, 109.4)	0.445

Data that do not conform to a normal distribution are represented by the median (interquartile range). Abbreviations: C_0_, trough concentrations of voriconazole; C_N_, trough concentrations of voriconazole-N-oxide; IL-6, interleukin-6; ALP, alkaline phosphatase; ALT, glutamic-pyruvic transaminase; AST, glutamic-oxalacetic transaminase; γ-GT, γ-glutamyl transferase; TBA, total bile acid; TBIL, total bilirubin; DBIL, direct bilirubin; IBIL, indirect bilirubin; eGFR, estimated glomerular filtration rate.

**FIGURE 1:**
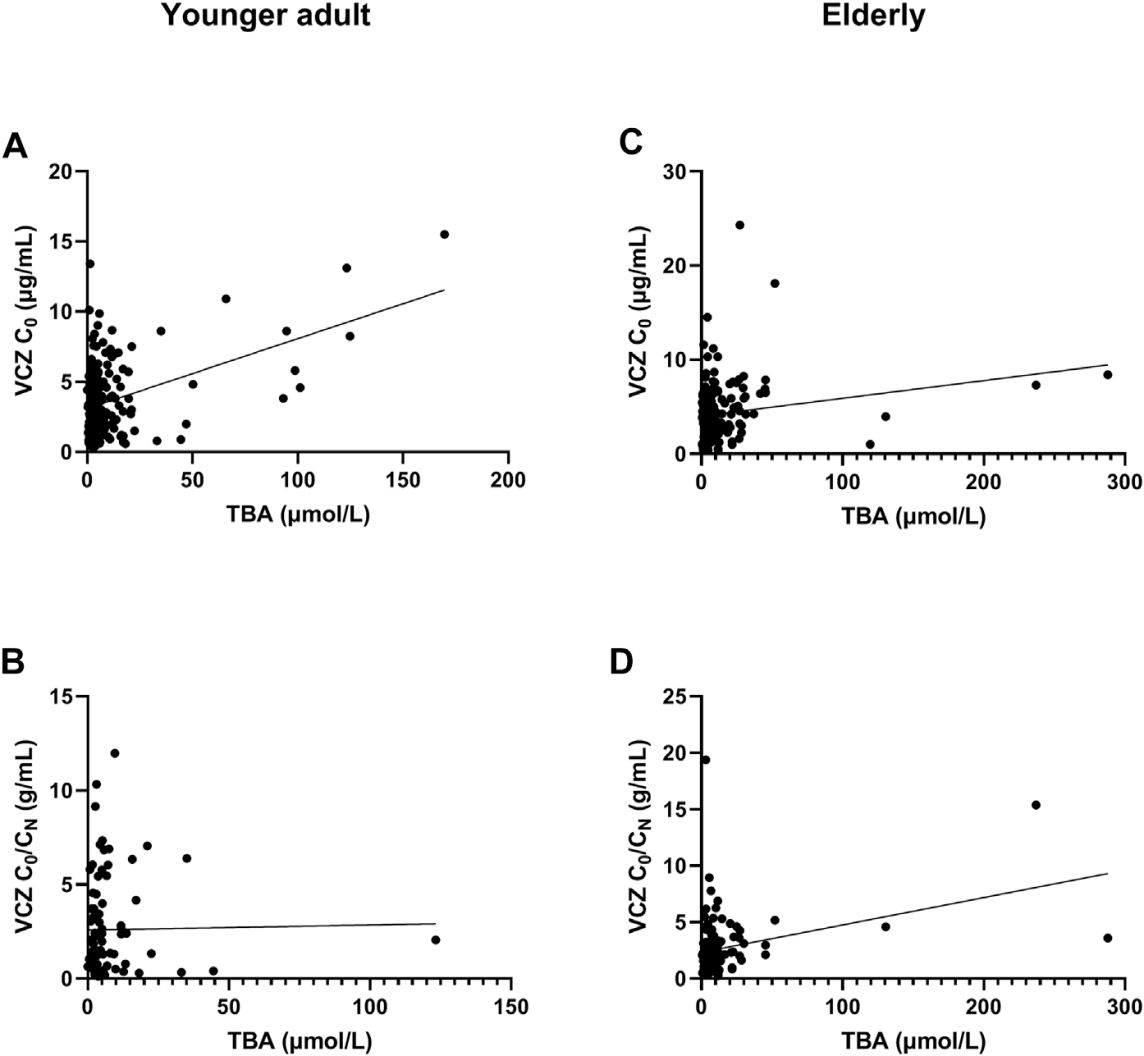
Association of total bile acid (TBA) level with voriconazole (VCZ) trough concentration (C_0_) and VCZ-to-VCZ N-oxide concentration ratio (C_0_/C_N_). **(A)**. The TBA level was positively associated with VCZ C_0_ in younger adult patients; **(B)**. There was no association between TBA level and VCZ C_0_/C_N_ in younger adult patients; **(C)**. There was a positive association between TBA level and VCZ C_0_ in elderly patients; **(D)**. The TBA level was positively associated with VCZ C_0_/C_N_ in elderly patients.

**FIGURE 2:**
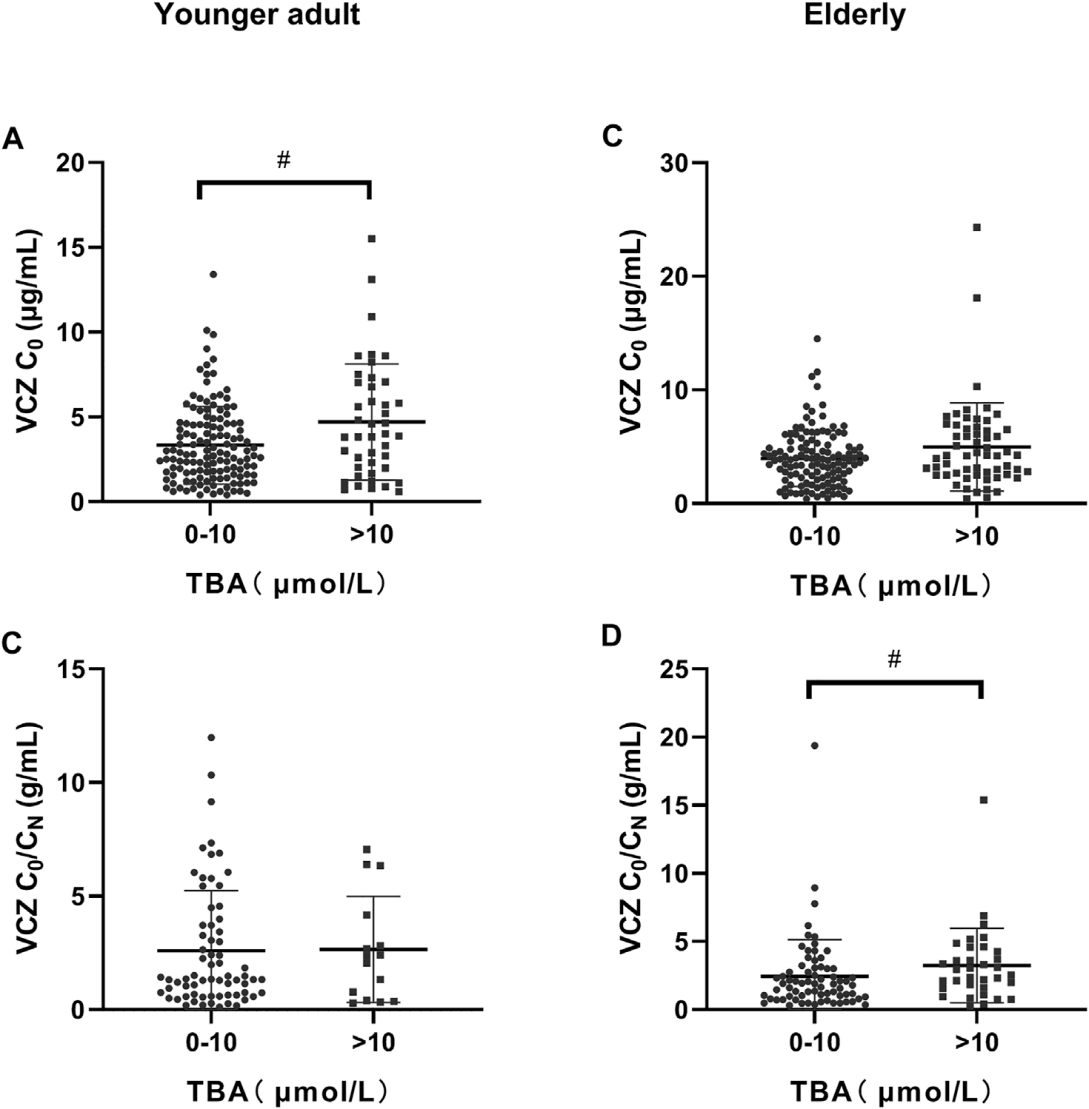
Distribution of voriconazole (VCZ) trough concentration (C_0_) and the VCZ-to-VCZ N-oxide concentration ratio (C_0_/C_N_) according to total bile acid (TBA) level. **(A)**. VCZ C_0_ in younger adult patients was significantly increased when TBA levels were higher than 10 μmol/L; **(B)**. VCZ C_0_/C_N_ in younger adult patients was similar when TBA levels were between 0 and 10 μmol/L and higher than 10 μmol/L; **(C)**. VCZ C_0_ in elderly patients was similar when TBA levels were between 0 and 10 μmol/L and greater than 10 μmol/L; **(D)**. VCZ C_0_/C_N_ in elderly patients increased significantly when TBA levels were higher than 10 μmol/L. ^#^ *p* < 0.05.

**FIGURE 3:**
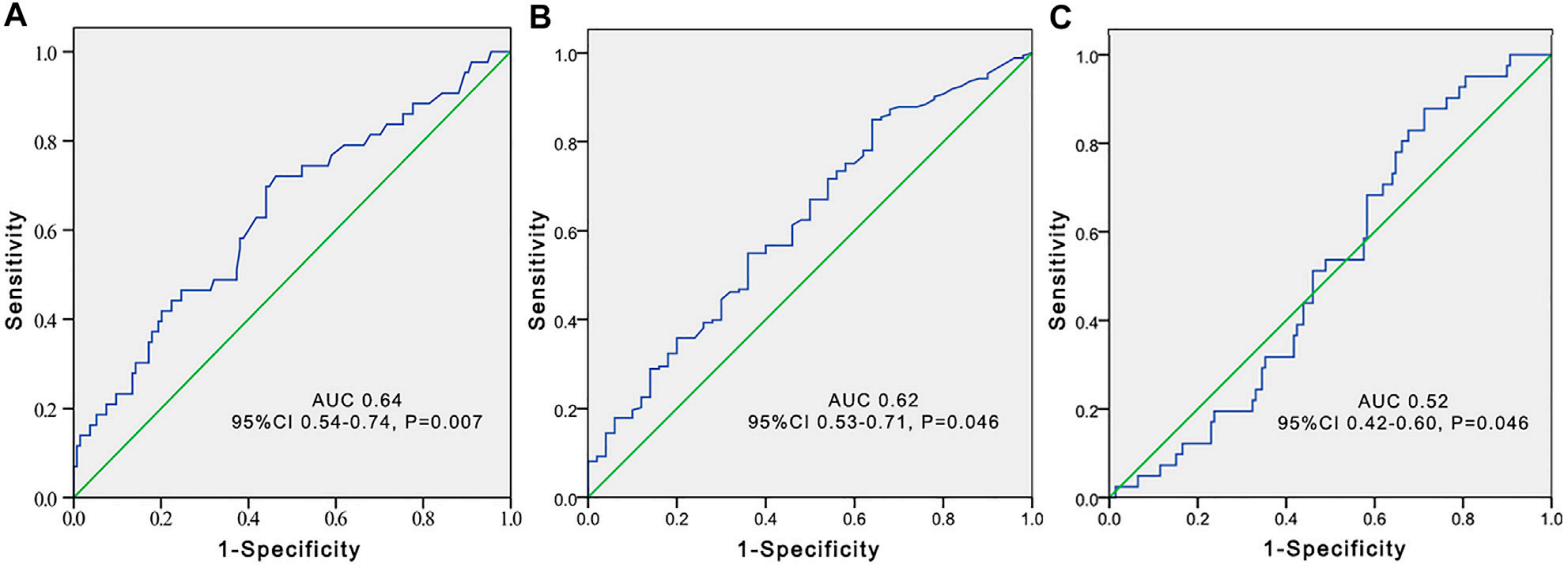
Receiver operating characteristic (ROC) curve to predict voriconazole trough concentrations greater than 5 μg/ml according to the total bile acid level **(A)**, platelet count **(B)**, and estimated glomerular filtration rate **(C)** in younger adult patients.

The independent influencing factors of VCZ C_0_ were levels of TBA and ALT and the use of PPIs. The independent influencing factors of VCZ C_0_/C_N_ were IL-6, age, DBIL, and TBA levels (Table 3). TBA values showed a positive association with VCZ C_0_ (*ρ* = 0.176, *p* = 0.019) but not with VCZ C_0_/C_N_ (*ρ* = 0.114, *p* = 0.305) (Figure 1). As shown in Figure 2, VCZ C_0_ increased significantly when TBA levels were higher than 10 μmol/L (*p* = 0.027). The analysis of the ROC curve indicated that TBA levels of ≥4.05 μmol/L, as well as the platelet count less than 31, increased the incidence of VCZ C_0_ greater than 5 μg/ml (95% CI = 0.54–0.74) (*p* = 0.007) (Figure 3). The ROC curve was not used for C_0_/C_N_ due to the lack of a clinically significant threshold.

**TABLE 3:** Influencing factor of VCZ C_0_ and C_0_/C_N_ in younger adult patients.

	VCZ C_0_				VCZ C_0_/C_N_		
Factor	OR (95% CI)	Standardized coefficients	*p*-Value	Factor	OR (95% CI)	Standardized coefficients	*p*-Value
Constant	5.074 (3.582, 6.565)	—	<0.001	Constant	−2.030 (−4.059, 0)	—	0.050
TBA	0.049 (0.029, 0.068)	0.428	<0.001	IL-6	0.002 (0.001, 0.002)	0.304	0.004
ALT	−0.016 (−0.029, −0.004)	−0.216	0.012	Age	0.089 (0.046, 0.132)	0.445	<0.001
PPI	−0.968 (−1.844, −0.092)	−0.180	0.031	DBIL	0.132 (0.067, 0.197)	0.684	<0.001
—	—	—	—	TBA	−0.057 (−0.108, −0.006)	−0.361	0.030

Abbreviations: TBA, total bile acid; ALT, glutamic-pyruvic transaminase; PPI, proton-pump inhibitor; DBIL, direct bilirubin.

### Elderly patients

A total of 143 elderly patients were included, with 234 VCZ C_0_ and 131 VCZ C_0_/C_N_. The primary baseline diseases were pneumonia, hypertension, and kidney disease. Thirty-four patients (23.8%) had negative fungi detection results. The proportion of men in the elderly cohort was higher than that in the younger adult cohort (*p* = 0.001). The route of VCZ administration in patients in the two cohorts was similar (*p* > 0.05). In contrast, the dose of VCZ in the elderly cohort was significantly lower than that in the younger adult cohort (*p* < 0.001) (Table 1).

VCZ C_0_ in the elderly cohort was significantly higher than that in the younger adult cohort (*p* < 0.05), while the VCZ C_0_/C_N_ ratio in the two cohorts was similar (*p* > 0.05). The percentages of ALP, ALT, AST, TBA, albumin, TBIL, and DBIL within the normal range were 57.9%, 67.5%, 58.4%, 67.8%, 73.4%, 71.6%, and 59.5%, respectively. The levels of IL-6, platelet count, hemoglobin, TBA, and urea nitrogen in the elderly cohort were significantly higher than those of the younger adult cohort (*p* < 0.05) (Table 2).

**FIGURE 4:**
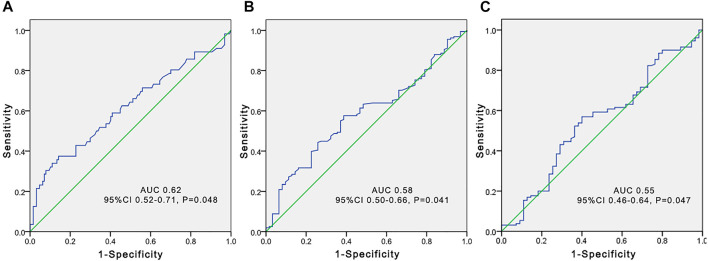
Receiver operating characteristic (ROC) curve to predict voriconazole trough concentrations greater than 5 μg/ml according to the total bile acid level **(A)**, platelet count **(B)**, and estimated glomerular filtration rate **(C)** in elderly patients.

The independent influencing factors of VCZ C_0_ were the levels of DBIL, albumin, and eGFR. The independent influencing factors of VCZ C_0_/C_N_ were eGFR, ALT, γ-GT, TBA, and platelet count (Table 4). The TBA level showed a positive association with VCZ C_0_ (*ρ* = 0.204, *p* = 0.006) and C_0_/C_N_ (*ρ* = 0.342, *p* < 0.001), respectively (Figure 1). VCZ C_0_/C_N_ significantly increased when TBA levels were higher than 10 μmol/L (*p* = 0.025) (Figure 2). ROC curve analysis indicated that when the TBA level ≥14.55 μmol/L, the incidence of a VCZ C_0_ greater than 5 μg/ml (95% CI = 0.52–0.71) (*p* = 0.048) increased (Figure 4).

**TABLE 4:** Influencing factor of VCZ C_0_ and C_0_/C_N_ in elderly patients.

VCZ C_0_					VCZ C_0_/C_N_				
Factor		OR (95% CI)	Standardized coefficients	*p*-Value	Factor		OR (95% CI)	Standardized coefficients	*p*-Value
Constant		10.112 (5.497, 14.726)	—	<0.001	Constant		3.736 (2.789, 4.683)	—	<0.001
DBIL		0.071 (0.027, 0.115)	0.307	0.002	eGFR		−0.014 (−0.022, −0.007)	−0.382	<0.001
Albumin		−0.160 (−0.288, −0.031)	−0.233	0.016	ALT		0.044 (0.026, 0.061)	0.462	<0.001
eGFR		−0.018 (−0.032, −0.003)	−0.235	0.016	γ-GT		−0.006 (−0.008, −0.003)	−0.398	<0.001
—		—	—	—	TBA		0.022 (0.004, 0.041)	0.230	0.019
—		—	—	—	Platelet count		−0.003 (−0.006, 0)	−0.224	0.026

Abbreviations: DBIL, direct bilirubin; eGFR, estimated glomerular filtration rate; ALT, glutamic-pyruvic transaminase; γ-GT, γ-glutamyl transferase; TBA, total bile acid.

## Discussion

VCZ-induced adverse reactions are generally considered the main reason for drug discontinuation and treatment failure associated with C_0_ (Jin et al., 2016; Hamada et al., 2020). Our previous study also showed a considerable number of VCZ C_0_ greater than 5 μg/ml, with a ratio of 23.4% in the younger adult cohort and 31.3% in the elderly cohort (Cheng et al., 2020). Therefore, it is crucial to investigate factors affecting VCZ metabolism. A significant correlation was found between VCZ C_0_ and age (Allegra et al., 2020; Bolcato et al., 2021). Niioka et al. found that older Japanese patients had higher VCZ C_0_/C_N_ ratios (Niioka et al., 2017). Age was also a predictor of VCZ trough levels >5 μg/ml (Chen et al., 2022). Therefore, we investigated the factors affecting VCZ C_0_ and C_0_/C_N_ in younger and elderly patients.

Our previous study found that IL-6 levels were associated with the VCZ C_0_/C_N_ ratio in both younger and elderly patients (r = 0.355, *p* = 0.003; r = 0.386, *p* = 0.001). Therefore, this study included IL-6 as an inflammatory marker. IL-6 can directly target liver cells and down-regulate CYP2C19 and CYP3A4 gene expression during inflammation (Li et al., 2014; Klein et al., 2015), affecting VCZ metabolism. Our results showed that IL-6 level was an independent influencing factor of VCZ C_0_/C_N_ in younger adults, which further confirmed the results of our previous study (Cheng et al., 2020; Liang et al., 2022).

Data on the effect of TBA on VCZ metabolism are limited. In the current study, TBA level was the independent influencing factor of VCZ C_0_ and C_0_/C_N_ in younger adult patients and the independent influencing factor of C_0_/C_N_ in older patients. TBA can effectively reflect the liver cell injury and the secretion and synthesis function of liver cells. TBA levels rise before the increase of bilirubin, which may partially explain our findings. Furthermore, the ROC curve identified the good predictive effects of TBA for VCZ C_0_ greater than 5 μg/ml. Our results indicate that TBA could be a good predictor of VCZ C_0_ in younger adult patients.

Platelets emerge as key players in inflammation and are key elements in the early phases of the inflammatory response (Nicolai and Massberg, 2020; Portier and Campbell, 2021). Accumulating evidence demonstrates that platelets contribute to the initiation and propagation of both local and systemic inflammatory processes (Manne et al., 2017). Since platelet count is routinely measured at our hospital, it was chosen as a key element in the inflammatory response. C-reactive protein (CRP) is an inflammatory marker commonly investigated in association with VCZ C_0_ and VCZ C_0_/C_N_ in IFI patients (Dote et al., 2016; Encalada Ventura et al., 2016; Veringa et al., 2017; Vreugdenhil et al., 2018). We did not include CRP in this study due to the limited CRP data in the elderly. We also omitted procalcitonin because its association with VCZ C_0_/C_N_ was insignificant in our previous study (Liang et al., 2022). Our results showed that platelet count was an independent influencing factor of VCZ C_0_/C_N_ in elderly individuals. Therefore, platelet count could be considered in patients on VCZ therapies.

Liver function is generally considered to influence VCZ metabolism. VCZ is bound to albumin. Decreased albumin levels increase the unbound fraction of VCZ (Vanstraelen et al., 2014). Serum albumin and γ-GT levels were significantly correlated with the VCZ clearance rate (Chantharit et al., 2020). This study found that albumin level was an independent influencing factor of VCZ C_0_, and the γ-GT level was an independent influencing factor of VCZ C_0_/C_N_ in elderly patients. Plasma TBIL concentration significantly influenced VCZ protein-protein binding (Vanstraelen et al., 2014). The TBIL level was associated with VCZ clearance in IFI patients with liver dysfunction (Tang et al., 2021). TBIL level was also considered an independent factor influencing VCZ C_0_ (Cheng et al., 2020; Zeng et al., 2020; Zhao Y. et al., 2021). However, our results showed that levels of DBIL but not TBIL influenced VCZ C_0_ and C_0_/C_N_. The liver is rich in a smooth endoplasmic reticulum (ER) equipped with enzymes that metabolize several drugs, including VCZ. DBIL is bioconverted to IBIL in the ER. DBIL levels may reflect the state of the ER and then exhibit an association with the metabolism of VCZ.

We found that eGFR was an independent influencing factor of VCZ C_0_ and VCZ C_0_/C_N_ in elderly individuals. Our results showed that the eGFR in the elderly cohort was lower than that in the younger adult cohort, indicating an impaired renal function in the elderly cohort. Although VCZ dose adjustment is not recommended for patients with renal impairment, we should still pay attention to its use in the elderly based on our results. Furthermore, the degree of inflammation in the elderly cohort was more severe than in the younger adult cohort, with impaired liver and kidney function. Therefore, the use of VCZ in elderly patients should be monitored.

CYP2C19, CYP3A4, and CYP2C9 enzymes metabolize PPIs. The combined use of PPIs with VCZ can affect VCZ concentration (Yan et al., 2018). PPIs also significantly affected VCZ C_0_ in younger adult patients in our study.

This study has several limitations. First, we did not include samples with VCZ C_0_ lower than 0.4 mg/L because the LLOQ of VCZ and VCZ N-oxide were both 400 ng/ml. Second, although the polymorphisms of CYP2C19*2 and *3 are critical for examining the pharmacokinetics of VCZ (Moriyama et al., 2017), the CYP2C19 genotypes were not assessed since testing is not routinely performed. Finally, this study had a relatively small sample size. A large multicenter, prospective study is needed to confirm our results.

In conclusion, we report for the first time that TBA, eGFR, and platelet count were associated with VCZ C_0_ and C_0_/C_N_. Furthermore, the TBA level had a good predictive effect on VCZ C_0_ in younger adult patients and may serve as a novel marker of VCZ metabolism. eGFR and platelet count should also be considered when using VCZ, especially in elderly patients.

## Data Availability

The raw data supporting the conclusions of this article will be made available by the authors, without undue reservation.
